# Association Between COVID-19 Exposure and Self-reported Compliance With Public Health Guidelines Among Essential Employees at an Institution of Higher Education in the US

**DOI:** 10.1001/jamanetworkopen.2021.16543

**Published:** 2021-07-21

**Authors:** Tracy L. Nelson, Bailey K. Fosdick, Laurie M. Biela, Hayden Schoenberg, Sarah Mast, Emma McGinnis, Michael C. Young, Lori Lynn, Scott Fahrner, Laura Nolt, Tina Dihle, Kendra Quicke, Emily N. Gallichotte, Emily Fitzmeyer, Greg D. Ebel, Kristy Pabilonia, Nicole Ehrhart, Sue VandeWoude

**Affiliations:** 1 Colorado School of Public Health, Colorado State University, Fort Collins; 2 Department of Health and Exercise Science, Colorado State University, Fort Collins; 3 Department of Statistics, Colorado State University, Fort Collins; 4 Human Performance Clinical Research Laboratory, Department of Health and Exercise Science, Colorado State University, Fort Collins; 5 Arthropod-Borne and Infectious Diseases Laboratory, Department of Microbiology, Immunology and Pathology, Colorado State University, Fort Collins; 6 Health Network, Colorado State University, Fort Collins; 7 Veterinary Diagnostics Laboratories, Colorado State University, Fort Collins; 8 Department of Microbiology, Immunology and Pathology, Colorado State University, Fort Collins; 9 Columbine Health Systems Center for Healthy Aging, Colorado State University, Fort Collins; 10 Department of Clinical Sciences, Colorado State University, Fort Collins; 11 One Health Institute, Colorado State University, Fort Collins

## Abstract

**Importance:**

Detailed analysis of infection rates paired with behavioral and employee-reported risk factors is vital to understanding how transmission of SARS-CoV-2 infection may be exacerbated or mitigated in the workplace. Institutions of higher education are heterogeneous work units that supported continued in-person employment during the COVID-19 pandemic, providing a test site for occupational health evaluation.

**Objective:**

To evaluate the association between self-reported protective behaviors and prevalence of SARS-CoV-2 infection among essential in-person employees during the first 6 months of the COVID-19 pandemic in the US.

**Design, Setting, and Participants:**

This cross-sectional study was conducted from July 13 to September 2, 2020, at an institution of higher education in Fort Collins, Colorado. Employees 18 years or older without symptoms of COVID-19 who identified as essential in-person workers during the first 6 months of the pandemic were included. Participants completed a survey, and blood and nasal swab samples were collected to assess active SARS-CoV-2 infection via quantitative reverse transcriptase–polymerase chain reaction (qRT-PCR) and past infection by serologic testing.

**Exposure:**

Self-reported practice of protective behaviors against COVID-19 according to public health guidelines provided to employees.

**Main Outcomes and Measures:**

Prevalence of current SARS-CoV-2 infection detected by qRT-PCR or previous SARS-CoV-2 infection detected by an IgG SARS-CoV-2 testing platform. The frequency of protective behavior practices and essential workers’ concerns regarding contracting COVID-19 and exposing others were measured based on survey responses.

**Results:**

Among 508 participants (305 [60.0%] women, 451 [88.8%] non-Hispanic White individuals; mean [SD] age, 41.1 [12.5] years), there were no qRT-PCR positive test results, and only 2 participants (0.4%) had seroreactive IgG antibodies. Handwashing and mask wearing were reported frequently both at work (480 [94.7%] and 496 [97.8%] participants, respectively) and outside work (465 [91.5%] and 481 [94.7%] participants, respectively). Social distancing was reported less frequently at work (403 [79.5%]) than outside work (465 [91.5%]) (*P* < .001). Participants were more highly motivated to avoid exposures because of concern about spreading the infection to others (419 [83.0%]) than for personal protection (319 [63.2%]) (*P* < .001).

**Conclusions and Relevance:**

In this cross-sectional study of essential workers at an institution of higher education, when employees reported compliance with public health practices both at and outside work, they were able to operate safely in their work environment during the COVID-19 pandemic.

## Key Points

**Question:**

What was the prevalence of SARS-CoV-2 infection and associated protective behaviors among essential employees at an institution of higher education during the first 6 months of the COVID-19 pandemic in the US?

**Findings:**

In this cross-sectional study of 508 essential employees, no cases of SARS-CoV-2 infection were verified by quantitative reverse transcriptase–polymerase chain reaction, and only 2 participants had measurable seroreactive IgG antibodies. High levels of handwashing and mask wearing were reported at work and outside work, and social distancing was reported significantly less often at work than outside work.

**Meaning:**

The findings suggest that compliance with protective behaviors both at work and outside work may be commensurate with the safe operation of complex work environments during a pandemic.

## Teaser

This cross-sectional study evaluates self-reported behaviors and cases of SARS-CoV-2 infection among essential in-person employees during the first 6 months of the COVID-19 pandemic at a US institution of higher education.

## Introduction

The spread of COVID-19, caused by SARS-CoV-2, has resulted in a global outbreak with more than 31 million documented infections and more than 560 000 deaths in the US alone.^1^ The pandemic resulted in closures of nonessential businesses nationwide as well as universities, schools, churches, restaurants, gyms, and many other workplaces.^1^ In the initial months of the pandemic, the extent of these closures varied by state and community, as did the association of these lockdowns with COVID-19 incidence.^2^ In communities in which marked decreases in case numbers occurred, considerations for safe workplace reentry were quickly adopted. Empirically derived models to minimize the risk of outbreaks in the workplace were lacking; therefore, states, communities, and businesses were forced to improvise to develop protocols that would allow a safe return to normal workforce productivity and function.

Although some instances of workplace-associated transmission of COVID-19 have been reported,^3,4,5,6^ these cases were largely associated with occupations with high exposure rates or workplace settings where public health safety guidelines are difficult to practice (eg, skilled nursing facilities, prisons, and meat processing plants). However, there is a paucity of information on the incidence of COVID-19 in workplace settings where outbreaks have not been confirmed. Among the most complex workplaces in which continued employment by so-called *essential employees* was supported were institutions of higher education (IHEs). These communities represented high-risk areas for disease transmission and outbreaks because they include residence halls and other congregate spaces, such as dining halls, locker rooms, lecture halls, and laboratories. Institutions of higher education may also include publicly accessed facilities, such as teaching hospitals or other service centers. Despite the occupational exposure risks, many IHE facilities and essential services did not completely shut down during stay-at-home phases of the COVID-19 pandemic.

Nearly all IHEs developed plans for managing students’ return to campus during fall 2020, with varying levels of success. For example, a recent study by Leidner et al^7^ revealed that US counties with large universities that included in-person instruction experienced a 56.2% increase in the incidence of COVID-19 during 21 days before through 21 days after classes started compared with a 17.9% decrease in counties in which universities operated remotely. Return-to-work models for IHEs are complex^8^ and, for the most part, have not been closely evaluated.

This study was therefore conducted to evaluate the association of a workforce reentry model with SARS-CoV-2 infection among essential workers at an IHE before students returned to campus in fall 2020. This institution included laboratories investigating SARS-CoV-2, a diagnostic laboratory certified by the Clinical Laboratory Improvement Amendments (CLIA) performing SARS-CoV-2 quantitative reverse transcriptase–polymerase chain reaction (qRT-PCR), a veterinary teaching hospital, and other research, teaching, and service activities that continued on campus throughout the period during which state shutdown orders were in place. We specifically investigated the prevalence of SARS-CoV-2 among asymptomatic individuals as well as previous exposure to the virus. We also report the frequency of protective behaviors at and outside the workplace as well as essential workers’ concerns regarding contracting COVID-19 and exposing others.

## Methods

### Study Design, Setting, and Participants

This cross-sectional study was conducted at an IHE in Fort Collins, Colorado. Employees of Colorado State University (CSU) were recruited to participate if they were identified by the human resources office to be essential in-person workers at the time of the stay-at-home (March 26 to April 26, 2020) and safer-at-home orders (April 27 to July 6, 2020) implemented by the State of Colorado Governor’s Office. The study was approved by the institutional review board at CSU. Written informed consent was obtained from participants before enrollment. This study followed the Strengthening the Reporting of Observational Studies in Epidemiology (STROBE) reporting guidelines for cross-sectional studies.

Initial invitations for participation in the study were sent on July 13, 2020, and the survey closed on September 2, 2020. Participants who had not enrolled were reinvited to participate at approximately 2-week intervals up to 3 times. Participants were first qualified through an online survey using Research Electronic Data Capture (REDCap), a secure, web-based software program designed to support data capture for research studies.^9,10^ Criteria for inclusion were age 18 years or older, ability to read and understand English, and not currently experiencing cough, shortness of breath or difficulty breathing, temperature >38 °C, chills or shaking with chills, muscle pain, new or worsening headaches, sore throat, or new loss of sense of taste or smell. Participants who reported these symptoms were advised to contact their primary care physicians and/or seek testing at 1 of the local county SARS-CoV-2 testing sites.

### Procedures

Participants were directed to a 90-question survey that included questions about work environment; COVID-19–protective behaviors, including social distancing, handwashing, and mask wearing; previous symptoms; exposures; testing; and perceptions of risk and health behaviors. Self-reported demographic information was also obtained for race/ethnicity and gender with options defined by the investigator including an “other” option (eAppendix in the Supplement). Race/ethnicity was assessed along with age, work unit, and other factors to examine potential moderators between exposure and outcome. After completing the survey, each participant was scheduled for a nasal mid-turbinate swab and venipuncture at the CSU Human Performance Clinical Research Laboratory (HPCRL) located on the CSU campus.

### Sample Processing

Blood and nasal swab samples were collected by research staff at the HPCRL. Samples were deidentified before they were sent to laboratories for analysis. Blood samples were obtained from the antecubital vein and placed into a serum collection tube. Research staff at the HPCRL were trained by a staff physician at the CSU Health and Medical Center on mid-turbinate nasal swab sample collection. Blood samples were centrifuged for 10 minutes at 1300*g*. Serum samples were separated into 0.5-mL aliquots and frozen at −20 °C until testing. Swab samples were placed in conical tubes containing 3 mL viral transport media (Hanks Balanced Salt Solution, 2% fetal bovine serum, 50 mg/mL gentamicin, 250 μg/mL amphotericin B–fungizone) and transported to the laboratory for analysis.

### Serologic and qRT-PCR Testing

Serum samples were tested for IgG antibodies seroreactive to SARS-CoV-2 antigens by the National Jewish Health CLIA Diagnostic Laboratory using an Abbott Architect IgG SARS-CoV-2 testing platform.^11,12^ RNA was extracted from nasal swab samples, and qRT-PCR was performed as described elsewhere.^13,14,15,16^ Samples with qRT-PCR reactivity were sent to a CSU CLIA-certified diagnostic laboratory for validation.^17^

### Statistical Analysis

Descriptive statistics and visualizations were created using the statistical program R, version 4.0.2 (R Project for Statistical Computing).^18^ All hypothesis tests were 1-sided and performed with statistical significance considered at *P* < .05, and 95% CIs are given. To compare reported protective behavior across types and locations, χ^2^ tests were performed. These tests were also used to compare participant concerns about exposing others to COVID-19 vs contracting it themselves. Mann-Whitney tests were used to assess whether protective behavior frequency (sometimes vs mostly/always), level of concern about contracting COVID-19 (not much/some vs quite a bit/very), and level of concern about exposing others to COVID-19 (not much/some vs quite a bit/very) varied by age. The associations between frequency of protective behavior and concerns were assessed using Spearman rank correlations. Logistic regression, along with Akaike information criteria measures of model fit and likelihood ratio tests, was used to quantify the associations of age and work unit with social distancing reports at work (sometimes vs mostly/always).

## Results

Of 1522 essential in-person employees invited to participate in this surveillance program, 15 did not meet eligibility criteria because they had 1 of the exclusionary symptoms; 508 (33.4%) of those who met eligibility criteria completed the study. These participants completed the survey and had samples evaluated for active infection using qRT-PCR and past infection by serologic testing. Enrolled employees represented all work units invited to participate.

Descriptive statistics for the 508 participants who completed all aspects of the study are provided in Table 1. Participants’ ages ranged from 18 to 70 years, with a mean (SD) age of 41.1 (12.5) years. A total of 305 participants (60.0%) were women, and 451 (88.8%) were non-Hispanic White individuals. Participants reported few chronic conditions with the exception of overweight or obesity (249 [51.2%]) based on self-reported height and weight.^19^

**Table 1.** Demographic, Health, and Unit Information Reported by the Study Participants

Characteristic	Participants, No. (%) (N = 508)
Work unit	
Veterinary teaching hospital	199 (39.2)
Facilities management	88 (17.3)
Housing and dining facilities	42 (8.3)
Residential dining	16 (3.1)
CSU Health and Medical Center	14 (2.8)
Laboratory animal resources	12 (2.4)
Other^a^	137 (27.0)
Age, y	
Mean (SD)	41.1 (12.5)
18-25	48 (9.4)
26-35	159 (31.3)
36-45	125 (24.6)
46-55	85 (16.7)
56-65	80 (15.8)
66-75	11 (2.2)
Gender	
Female	305 (60.0)
Male	200 (39.4)
Other^b^	3 (0.6)
Hispanic/Latino	
Yes	46 (9.1)
Race/ethnicity	
White	451 (88.8)
Asian	30 (5.9)
American Indian or Alaskan Native	17 (3.3)
Black or African American	8 (1.6)
Native Hawaiian or other Pacific Islander	4 (0.8)
Other^c^	8 (1.6)
BMI^d^	
Underweight	9 (1.8)
Normal	228 (46.8)
Overweight	142 (29.2)
Obese	107 (22.0)
Health behavior or condition	
Exercise^e^	388 (76.4)
Asthma	62 (12.2)
Diabetes	11 (2.2)
History of blood clots	16 (3.1)
High blood pressure	52 (10.2)
Allergies	250 (49.2)

Abbreviation: BMI, body mass index (calculated as weight in kilograms divided by height in meters squared).

^a^ Other work units included research laboratories, central receiving department, office of campus police, and university housing.

^b^ Other responses included everything except male or female.

^c^ Other responses included individuals that identified as belonging to a racial group other than those listed. Multiple races could be selected.

^d^ Underweight was defined as a BMI less than 18.5; normal, 18.5 to 24.9; overweight, 25.0 to 29.9; and obese as 30 or greater.

^e^ Number responding yes to the question “Do you get at least 150 minutes of moderate exercise (brisk walking, slow biking, dancing) or 75 minutes of vigorous exercise (running or jogging, swimming, basketball, tennis) per week?”

Most participants (406 [79.9%]) reported spending more than 20 hours per week on the CSU campus from March 15, 2020, until they took the survey. A total of 335 participants (65.9%) reported spending most of their time at work with employees from the same unit (eTable 1 in the Supplement).

Participants reported practicing protective behaviors while at work and outside work. Table 2 shows that while at work, most employees reported mostly or always wearing a face mask (496 [97.8%; 95% CI, 92.2%-96.4%]), whereas fewer reported mostly or always practicing social distancing (403 [79.5%; 95% CI, 75.7%-82.9%]) (χ^2^ = 332; *df* = 4; *P* < .001 for test of differences across behaviors). Outside work, the percentages were similar, with 465 (91.5%; 95% CI, 88.7%-93.7%) reporting handwashing and 481 (94.7%; 95% CI, 92.3%-96.4%) reporting mask wearing always or mostly, except that 465 respondents (91.5%; 95% CI, 88.7%-93.7%) reported practicing social distancing (χ^2^ = 37; *df* = 4; *P* < .001 for test of differences across behaviors). The frequency of social distancing at work and outside work was significantly different (χ^2^ = 80; *df* = 2; *P* < .001). Overall, protective behavior varied little by age except for social distancing, with younger age groups reporting less social distancing at work (*U*, 17 223; 1-sided *P* = .003) (eFigure 1 in the Supplement). Specifically, 100% of those older than 65 years reported high levels of social distancing at work, whereas only 83.3% (95% CI, 70.4%-91.3%) of employees aged 18 to 25 years reported mostly or always social distancing at work. There were few differences in protective behavior by gender (eFigure 2 in the Supplement) but greater variation in these behaviors among work units (eFigure 3 in the Supplement). Levels of social distancing in the workplace varied significantly by work unit (χ^2^ = 27.8; *df* = 6; *P* < .001) and by age group (χ^2^ = 15.5; *df* = 5; *P* = .009), with work unit explaining more variability than age (Akaike information criteria, 458.2 vs 470.0). After accounting for work unit, age remained significantly associated with social distancing levels at work (likelihood ratio test deviance, 11.45; *df* = 5; *P* = .04).

**Table 2.** Frequency of Protective Behaviors Among Participants While at Work and Outside Work

Behavior	Participants, No. (%)^a^		
	Always	Mostly	Sometimes
At work			
Social distance	200 (39.4)	203 (40.0)	104 (20.6)
Wash hands	397 (78.3)	83 (16.4)	27 (5.3)
Face mask	453 (89.3)	43 (8.5)	11 (2.2)
Outside work			
Social distance	340 (66.9)	125 (24.6)	43 (8.4)
Wash hands	356 (70.1)	109 (21.5)	43 (8.4)
Face mask	420 (82.7)	61 (12.0)	27 (5.3)

^a^ The percentages in each row sum to 100.

Employees reported being more concerned (ie, very to quite a bit) about exposing others to COVID-19 (419 [83.0%; 95% CI, 79.3%-86.1%]) than about contracting COVID-19 (319 [63.2%; 95% CI, 58.8%-67.4%]; χ^2^ = 49; *df* = 1; *P* < .001) (Table 3). The level of concern about contracting COVID-19 appeared to vary by age, with older adults being most concerned (*U*, 28 624; *P* = .51); there was little variation by age with regard to concern about exposing others, with most people reporting being very concerned (*U*, 20 164; *P* = .08) (eFigure 4 in the Supplement).

**Table 3.** Frequency of Participant Concerns About Contracting and Exposing Others to COVID-19

Concern	Participants, No. (%)^a^			
	Very	Quite a bit	Some	Not much
Concern about contracting COVID-19	147 (29.1)	172 (34.1)	120 (23.8)	66 (13.0)
Concern about exposing others to COVID-19	308 (61.0)	111 (22.0)	69 (13.7)	17 (3.3)
Important to know if previously exposed	236 (46.6)	127 (25.1)	127 (25.1)	16 (3.2)

^a^ The percentages in each row sum to 100.

**Figure 1.**
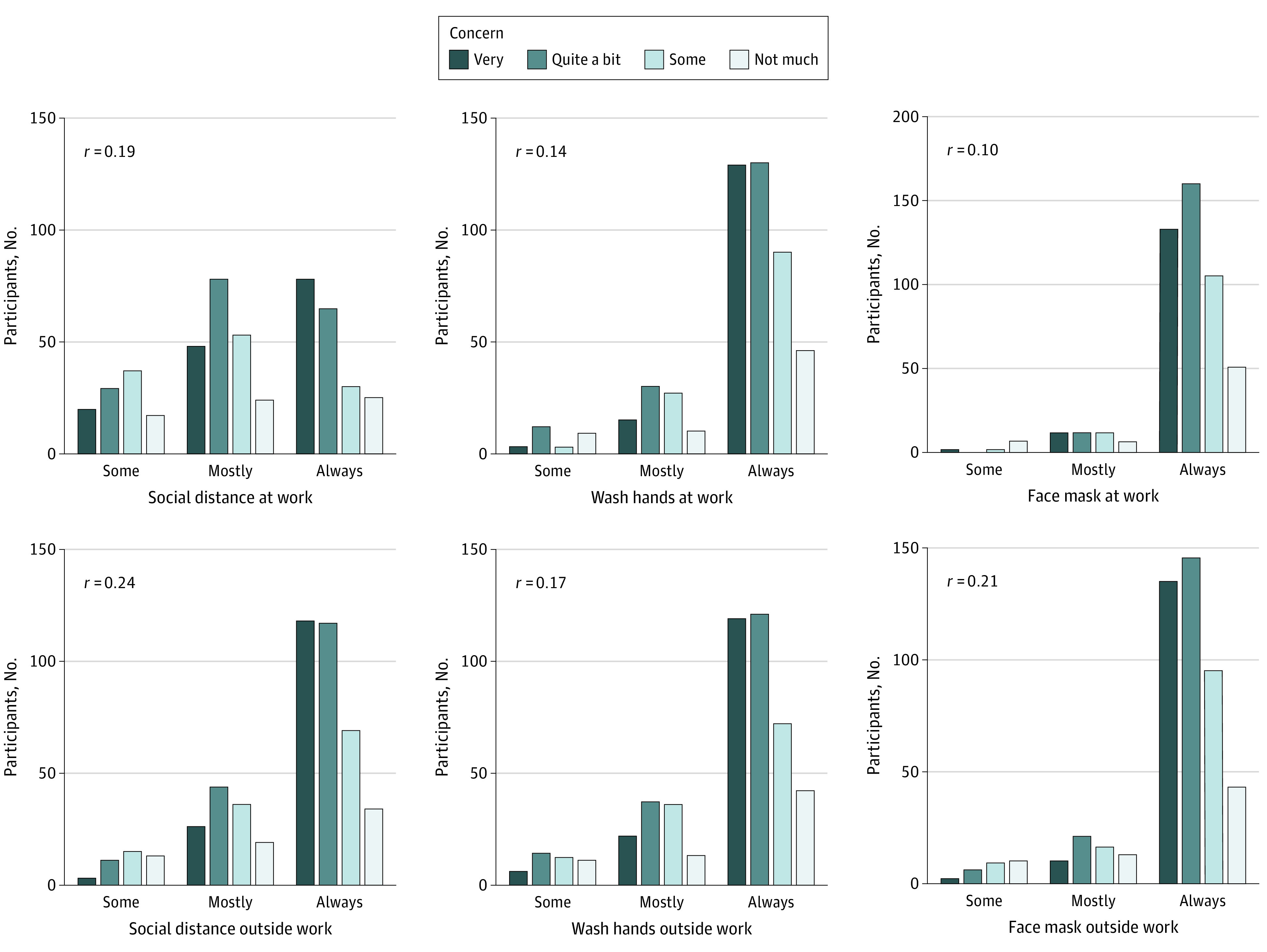
Frequency of Protective Behaviors by Level of Concern About Contracting COVID-19 All Spearman rank correlations were significant.

**Figure 2.**
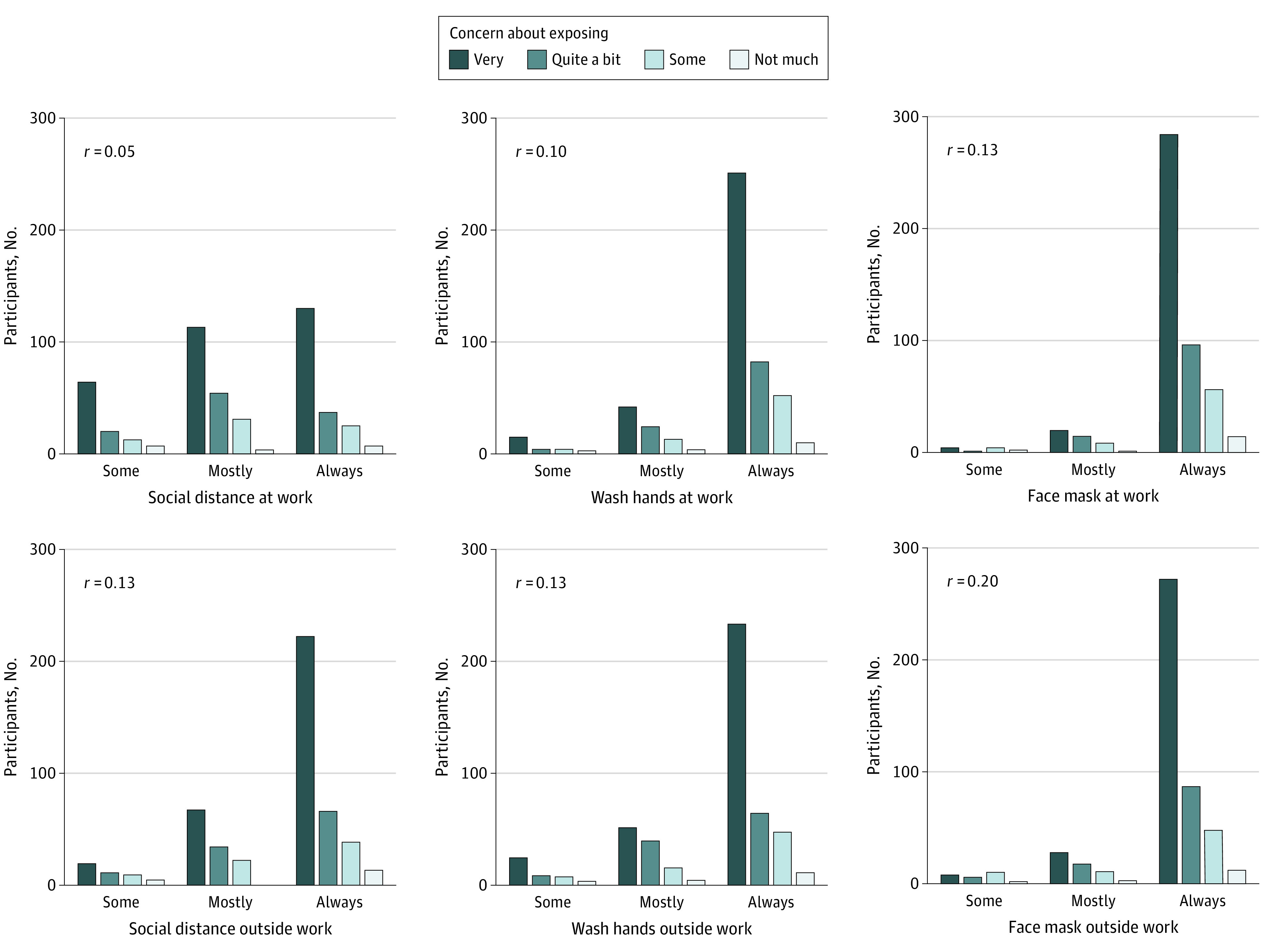
Frequency of Protective Behaviors by Level of Concern About Exposing Others to COVID-19 All Spearman rank correlations were significant except social distancing at work.

Protective behaviors varied by concern about contracting COVID-19. Those who reported always social distancing, washing their hands, and wearing a face mask at work and outside work were more concerned about contracting COVID-19 than were those who reported these behaviors some of the time (*r*, 0.10-0.24) (Figure 1). Similarly, protective behaviors were practiced more frequently by individuals with substantial concerns about exposing others to COVID-19 (*r,* 0.05-0.20) (Figure 2), although social distancing at work was not significantly correlated with concern about exposing others (*r*, 0.5; *P* = .26).

### Nasal Swab and Serologic Test Results

A total of 508 nasal swab samples were tested using qRT-PCR; results were negative for 502 and inconclusive for 6 in a laboratory-based screening test. These samples were sent to the CSU CLIA-certified diagnostic laboratory for confirmatory testing, by which all test results were confirmed to be negative (0 positive qRT-PCR results among 508 tests). Of 508 serum samples tested, 2 were seroreactive for IgG antibodies, indicating previous SARS-CoV-2 exposure, resulting in a 0.4% (95% CI, 0.1%-1.4%) seroprevalence estimate. Of the 2 workers who were seropositive for SARS-CoV-2, 1 reported having a previous positive qRT-PCR test result on the deidentified survey; the other individual had not been previously tested but reported previous symptoms of COVID-19.

Overall, most employees reported no known contact with persons who had positive test results (467 [91.9%]) or contact with a symptomatic individual (440 [86.6%]). A total of 65 participants (12.8%) reported having had a previous qRT-PCR test, and 23 (4.5%) reported a previous serologic test. Despite the low rates of previous testing or positivity, 143 participants (28.1%) reported having had previous COVID-19–like symptoms (eTable 2 in the Supplement).

## Discussion

Institutions of higher education represent a heterogeneous setting in which to evaluate exposure of a diverse population of employees who reported to work during the first 6 months of the COVID-19 pandemic. The first step in our IHE workforce reentry model included inviting all essential workers without symptoms to undergo SARS-CoV-2 testing using qRT-PCR. Our goal was to identify asymptomatic workers and temporarily remove them from the workforce. We did not identify any cases of SARS-CoV-2 infection in this asymptomatic group of employees despite most of these employees working on campus for more than 20 hours per week. Furthermore, only 2 participants tested positive for antibodies, indicating prior exposure; however, 41 reported having been exposed to someone who tested positive, and 143 reported experiencing COVID-19 symptoms in the past (eTable 2 in the Supplement). This low rate of seroprevalence (0.4%; 95% CI, 0.1%-1.4%) was approximately 10% of the estimated rate of seroprevalence (3.6%) in the county at the time of this study.^20^

Although the county case rates were relatively low (approximately 20 per day) at the time of this study, CSU faculty and staff continued to record low case rates (<2 per day) during the period when county case rates were the highest (approximately 200 per day) in early December 2020.^20,21^ Given the recommendations for social distancing, handwashing, and use of face masks to decrease virus transmission,^22,23,24^ these low numbers may be attributed to the high percentage of employees who reported participating in protective behaviors and generally feeling responsible for protecting their own health and that of others (Figure 1 and Figure 2). We found that 91% to 98% of employees reported mostly or always regularly washing their hands and wearing a face mask (Table 2). Social distancing mostly or always outside work was also reported by a high percentage of participants (91.5%; 95% CI, 88.7%-93.7%) (Table 2). Our findings are similar to those of a study^25^ reporting that student behaviors outside campuses were most responsible for the spread of SARS-CoV-2 infection on campuses, and with proper precautions, the prevalence of spread of the infection was lower in classrooms and other formal spaces. These findings suggest that an IHE can safely operate when employees responsibly practice public health guidelines for infection control both at work and outside work.

A small but substantial percentage of employees reported social distancing at work mostly or always (79.5%; 95% CI, 75.7%-82.9%) (Table 2). This finding suggests that work conditions may not always be conducive to practicing social distancing behaviors, and we did find that differences in social distancing practice varied by work unit (eFigure 3 in the Supplement).

We also found that social distancing at work varied by employees’ age, with 100% of those older than 65 years reporting high levels of social distancing at work compared with 83.3% (95% CI, 70.4%-91.3%) of those aged 18 to 25 years (eFigure 1 in the Supplement). These results suggest that those with greater risk of severe disease were more likely to conform to social distance recommendations. Because mask wearing and handwashing were independently controlled and more easily managed during workplace interactions, these compliance measures were likely easier to maintain than social distancing. However, work unit explained more of the variation in social distancing than age, suggesting that workplace situations may have occurred in which social distancing was not always possible because of space limitations or interactions with the public. For example, workers in the veterinary clinic and facilities support staff needed to occasionally come into close contact with other workers to complete essential tasks.

Adherence to protective behaviors both in the workplace and outside work correlated with reported concerns about contracting COVID-19 (Figure 1). Individuals who reported always social distancing, washing their hands, and wearing a face mask at work and outside work also reported being most concerned with contracting COVID-19. These findings are similar to those reported in a study^26^ of a US sample during the first week of the pandemic in which social distancing and handwashing were most strongly associated with individuals’ perceived likelihood of becoming infected.

In the present study, employees were more concerned about exposing others to COVID-19 than about contracting COVID-19 (83.0% [95% CI, 79.3%-86.1%] vs 63.2% [95% CI, 58.8%-67.4%]), and this concern was associated with protective behaviors (Figure 2). Such concern can be described as a prosocial behavior, or a behavior that is helpful and intended to promote social acceptance.^27^ A recent study^28^ conducted in Sweden found that those scoring higher on a measure of prosocial behavior were more likely to follow physical distancing guidelines, stay at home when sick, and buy face masks during the COVID-19 pandemic. Multiple motivations for prosocial behavior have been described including empathy and shared social identity, which can be built through effective leadership.^29^ Prosocial behavior has been shown to lead to greater positive affect, meaningfulness, empathy, and social connectedness,^30^ which may influence employees’ desire to protect their coworkers from an infectious virus (or any other threat) as well as overall morale.

We believe that future studies should examine the practice of protective behaviors outside the workplace because even if protective behaviors are regulated or expected at work, exposure to SARS-CoV-2 infection outside work can increase the risk of spread in the workplace. Furthermore, prosocial behavior appeared to be a motivator for practicing safe behaviors and should be fostered by unit and institutional leaders because it may help such institutions remain open.

### Limitations

This study has limitations. We used self-reported data, which could have resulted in response bias for reported protective behaviors. Because this was a cross-sectional study, we could not determine temporality between protective behaviors and COVID-19 outcomes. However, given that no cases of COVID-19 were detected and only 2 participants were seropositive for SARS-CoV-2, previous exposure was unlikely to have been associated with protective behaviors and protective behaviors may have been associated with the low level of disease in this population. Nonresponse bias, whereby those who were invited and did not participate differed from those who did, might have been present. However, the participation rate (33.7%) and the number of respondents (508) were substantial and helped to mitigate this concern.

## Conclusions

This cross-sectional study revealed low levels of active or previous exposure to SARS-CoV-2 infection in a large cohort of employees whose job types required them to work on a university campus while state restrictions were in place during the COVID-19 pandemic. The absence of cases of COVID-19 and the low seroprevalence of SARS-CoV-2 may have been associated with the high rates of protective behaviors by employees at work and outside work. Although these results reflect the experience at 1 IHE, we believe these results could be generalized to other IHEs and complex work environments.

## Notes

Supplement. **eTable 1.** Participants reported contact with others while at the workplace, ranging from none of the time to >50% of the time **eTable 2.** Participants reported history of exposure, testing and symptoms for COVID-19 **eFigure 1.** Frequency of protective behaviors at and outside the workplace by age **eFigure 2.** Frequency of protective behaviors at and outside the workplace by gender **eFigure 3.** Frequency of protective behaviors at and outside the workplace by work unit **eFigure 4.** Concerns for contracting and exposing others to COVID-19 by age **eAppendix.** 90-Question Survey
