# Supplementary material for: Association Between COVID-19 Exposure and Self-reported Compliance With Public Health Guidelines Among Essential Employees at an Institution of Higher Education in the US
Source: JAMA Netw Open. 2021 Jul 21;4(7):e2116543. doi: 10.1001/jamanetworkopen.2021.16543 (PMC8295736; doi:10.1001/jamanetworkopen.2021.16543)
Supplement: Supplement. — eTable 1. Participants reported contact with others while at the workplace, ranging from none of the time to >50% of the time eTable 2. Participants reported history of exposure, testing and symptoms for COVID-19 eFigure 1. Frequency of protective behaviors at and outside the workplace by age eFigure 2. Frequency of protective behaviors at and outside the workplace by gender eFigure 3. Frequency of protective behaviors at and outside the workplace by work unit eFigure 4. Concerns for contracting and exposing others to COVID-19 by age eAppendix. 90-Question Survey [file jamanetwopen-e2116543-s001.pdf]

Supplementary Online Content

Nelson TL, Fosdick BK, Biela LM, et al. Association between COVID-19 exposure and self-reported compliance with public health guidelines among essential employees at an institute of higher education in the US. *JAMA Netw Open*. 2021;4(7):e2116543. doi:10.1001/jamanetworkopen.2021.16543

eTable 1. Participants reported contact with others while at the workplace, ranging from none of the time to >50% of the time

eTable 2. Participants reported history of exposure, testing and symptoms for COVID-19

eFigure 1. Frequency of protective behaviors at and outside the workplace by age.

eFigure 2. Frequency of protective behaviors at and outside the workplace by gender

eFigure 3. Frequency of protective behaviors at and outside the workplace by work unit

eFigure 4. Concerns for contracting and exposing others to COVID-19 by age

eAppendix. 90-Question Survey

This supplementary material has been provided by the authors to give readers additional information about their work.

© 2021 Nelson TL et al. *JAMA Network Open*.

eTable 1. Participants reported contact with others while at the workplace, ranging from none of the time to ≥50% of the time.

Contact in the workplace	None	<50%	≥50%
Employees, same unit, No. (%)	10 (2)	163 (32)	335 (66)
Employees, outside unit, No. (%)	141 (28)	320 (63)	47 (9)
General public, No. (%)	193 (38)	286 (56)	29 (6)
Students, No. (%)	169 (33)	263 (52)	76 (15)

Note: The percentages in each row sum to 100.

eTable 2: Participants reported history of exposure, testing and symptoms for COVID-19.

	Yes	No	Don't Know
Contact with COVID-19 positive individual, No. (%)	41 (8.1)	299 (58.9)	168 (33)
Contact with individual showing symptoms, No. (%)	68 (13.4)	295 (58.1)	145 (29)
Previous PCR test for COVID-19, No. (%)	65 (12.8)	443 (87.2)	
Previous Positive PCR test, No.	1		
Previous Serology test for COVID-19 antibodies, No. (%)	23 (4.5)	485 (95.5)	
Previous Positive Serology test, No.	1		
Previous COVID-like symptoms, No. (%)	143 (28.1)	365 (71.9)	

© 2021 Nelson TL et al. *JAMA Network Open*.

eFigure 1: Frequency of protective behaviors at and outside the workplace by age.

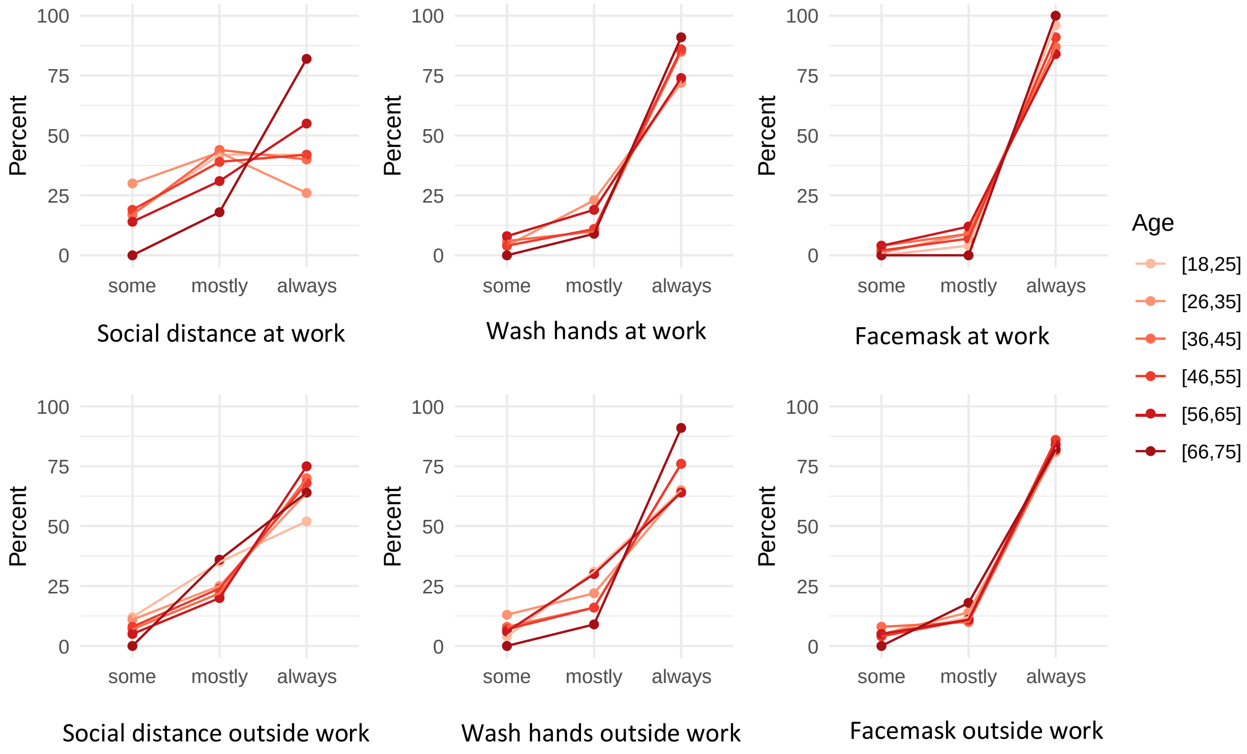

© 2021 Nelson TL et al. *JAMA Network Open*.

eFigure 2: Frequency of protective behaviors at and outside the workplace by gender.

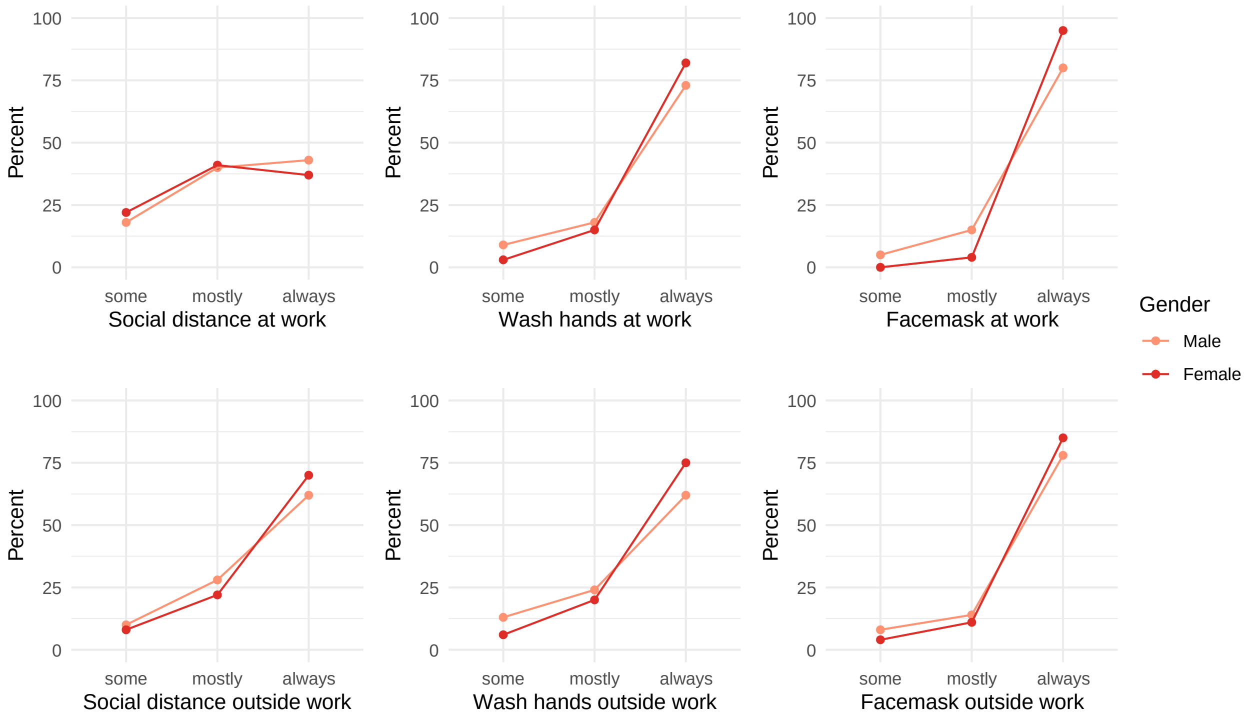

© 2021 Nelson TL et al. *JAMA Network Open*.

eFigure 3: Frequency of protective behaviors at and outside the workplace by work unit.

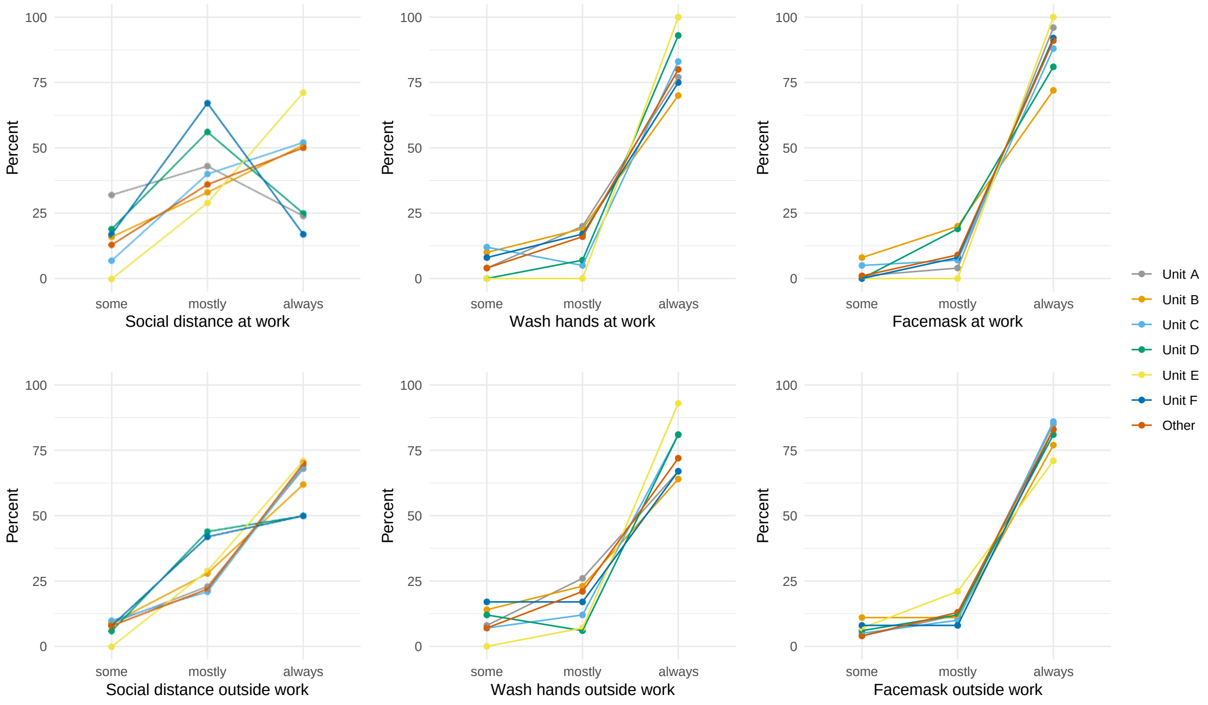

© 2021 Nelson TL et al. *JAMA Network Open*.

eFigure 4: Concerns for contracting and exposing others to COVID-19 by age.

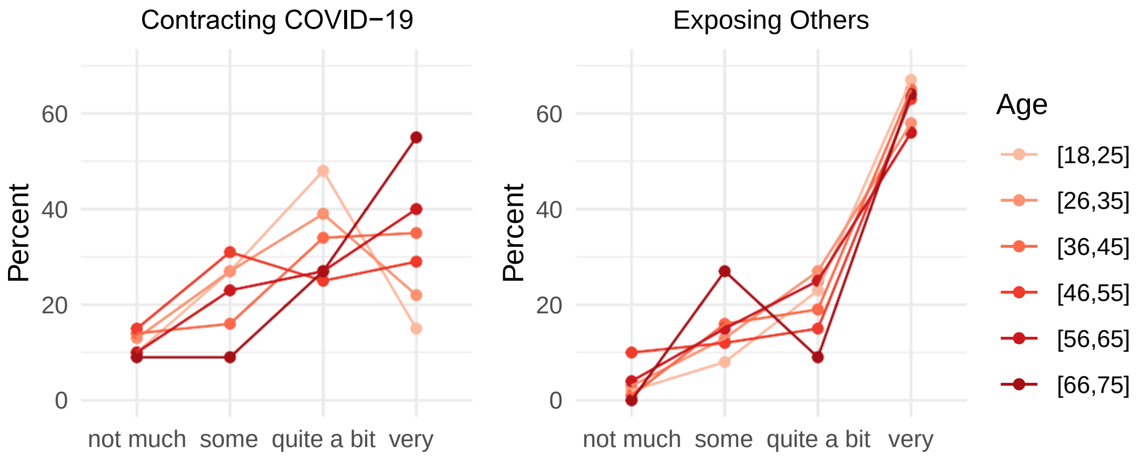

© 2021 Nelson TL et al. *JAMA Network Open*.

eAppendix. COVID Questionnaire

Please complete the survey below.

Thank you!

Thank you for participating in the RESTARtT study. Your responses will help us better understand COVID-19 exposure and risk factors. Please answer the following questions honestly and completely. All surveys will be anonymous and data will be analyzed at the group level to protect your privacy and identity.

Section 1: Work Environment

What unit or department do you work for at CSU?	◯ Housing and Dining Facilities<br>◯ University Housing ◯ Residential Dining<br>◯ Facilities Management<br>◯ Laboratory Animal Resources<br>◯ Health Network Medical<br>◯ Veterinary Teaching Hospital<br>◯ Research Lab ◯ Other
What department do you work for?	______________________
Do you conduct research pertaining to COVID-19?	◯ Yes ◯ No
Since March 15th, how many hours per week, on average, have you been on CSU campus while you have been working?	◯ < 5 hours/week<br>◯ 5-9 hours/week<br>◯ 10-19 hours/week<br>◯ 20-30 hours/week<br>◯ >30 hours/week

Please answer the following questions about working with other people from March 15th through the present.

Since March 15th, on average, how much in-person time do you spend with other employees in the same unit while working on the CSU campus?	◯ None<br>◯ Less than half of my work hours<br>◯ More than half of my work hours
Since March 15th, on average, how much in-person time do you spend with students on the CSU campus?	◯ None<br>◯ Less than half of my work hours<br>◯ More than half of my work hours
Since March 15th, on average, how much in-person time do you spend with the general public (for example, clients, contractors) while working on the CSU campus?	◯ None<br>◯ Less than half of my work hours<br>◯ More than half of my work hours
Since March 15th, on average, how much in-person time do you spend with CSU employees outside your unit while working on the CSU campus?	◯ None<br>◯ Less than half of my work hours<br>◯ More than half of my work hours
Since March 15th, on average, how much in-person time do you spend with people, not in your household, while not at work?	◯ None<br>◯ Less than 1 hour per day<br>◯ Between 1 and 3 hours per day<br>◯ More than 3 hours per day

© 2021 Nelson TL et al. *JAMA Network Open*.

Since March 15th, how have you protected yourself from the coronavirus (COVID-19) at work?

I social distance at least 6 ft: Never Sometimes Always
(Place a mark on the scale above)

I use some type of face mask: Never Sometimes Always
(Place a mark on the scale above)

I frequently wash my hands: Never Sometimes Always
(Place a mark on the scale above)

Other ways I have protected myself from the coronavirus at work. ______________________

Since March 15th, how have you protected yourself from the coronavirus (COVID-19) outside of your home, when you are not at work?

I social distance at least 6 ft: Never Sometimes Always
(Place a mark on the scale above)

I use some type of face mask: Never Sometimes Always
(Place a mark on the scale above)

I frequently wash my hands: Never Sometimes Always
(Place a mark on the scale above)

Other ways I have protected myself from the coronavirus outside of my home, when not at work. ______________________

Section II: Past symptoms:

Since around February 1st, 2020 have you experienced any of the following symptoms?

Cough (new onset or worsening of chronic cough) ◯ Yes ◯ No

How would you classify your cough? ◯ Mild ◯ Moderate ◯ Severe

Approximate date started ______________________

How many days did it last? ______________________

Shortness of breath or difficulty breathing ◯ Yes ◯ No

How would you classify your shortness of breath? ◯ Mild ◯ Moderate ◯ Severe

Approximate date started ______________________

© 2021 Nelson TL et al. *JAMA Network Open*.

How many days did it last?	__________________________________
Fever >100.4F (38.4C)	◯ Yes ◯ No
How many days did it last?	__________________________________
Chills/shaking with chills	◯ Yes ◯ No
How would you classify your chills?	◯ Mild ◯ Moderate ◯ Severe
Approximate date started	__________________________________
How many days did it last?	__________________________________
Muscle pain	◯ Yes ◯ No
How would you classify your muscle pain?	◯ Mild ◯ Moderate ◯ Severe
Approximate date started	__________________________________
How many days did it last?	__________________________________
Headache (new onset or worsening of chronic headaches)	◯ Yes ◯ No
How would you classify your headache?	◯ Mild ◯ Moderate ◯ Severe
Approximate date started	__________________________________
How many days did it last?	__________________________________
Sore throat	◯ Yes ◯ No
How would you classify your sore throat?	◯ Mild ◯ Moderate ◯ Severe
Approximate date	__________________________________
How many days did it last?	__________________________________
New loss of sense of taste or smell	◯ Yes<br>◯ No
How would you classify this?	◯ Mild ◯ Moderate ◯ Severe

© 2021 Nelson TL et al. *JAMA Network Open*.

Approximate date started

____

How many days did it last?

____

Other symptoms not listed?

____

Section III: Exposure, Testing History and Perceptions

How many people live with you NOT including yourself?
- ◯ 0
- ◯ 1
- ◯ 2
- ◯ >2

Do any of the individuals living with you work with COVID-19 patients?
- ◯ Yes
- ◯ No

Are any of the individuals living with you first responders?
- ◯ Yes
- ◯ No

Have you had any kind of in-person contact with someone who tested positive for coronavirus (COVID-19)?
- ◯ Yes
- ◯ No
- ◯ Do not know

Does this person live with you?
- ◯ Yes
- ◯ No

When did this contact occur?

____

Was this contact within 6 feet for an extended period of time?
- ◯ Yes
- ◯ No
- ◯ Do not know

How long were you within 6 feet of this individual?

____

Were you wearing a facemask during the time of contact?
- ◯ Yes
- ◯ No
- ◯ Do not know

Was the infected person wearing a face mask?
- ◯ Yes
- ◯ No
- ◯ Do not know

Have you had any kind of in-person contact with someone who had symptoms of the coronavirus (COVID-19)?
- ◯ Yes
- ◯ No
- ◯ Do not know

Does the person live with you?
- ◯ Yes
- ◯ No

© 2021 Nelson TL et al. *JAMA Network Open*.

When did this contact occur?	________________________________
Was this contact within 6 feet for an extended period of time?	◯ Yes<br>◯ No<br>◯ Do not know
How long were you within 6 feet of this individual?	________________________________<br>(For example 30min)
Were you wearing a mask during the time of contact?	◯ Yes ◯ No ◯ Do not know
Was the symptomatic individual wearing a face mask?	◯ Yes<br>◯ No<br>◯ Do not know
Have you had a nasal swab to test for the coronavirus (COVID-19)?	◯ Yes<br>◯ No
What were the results of your test?	◯ Positive<br>◯ Negative<br>◯ Do not know
What was the date of your test?	________________________________
If tested multiple times what was the date of your second test?	________________________________
Have you been tested for antibodies to the coronavirus (COVID-19)?	◯ Yes<br>◯ No
What were the results of your antibody test?	◯ Positive<br>◯ Negative<br>◯ Do not know
What was the date of your antibody test?	________________________________
If you have not had the coronavirus, please rate on the scale how concerned you are about getting the coronavirus (COVID-19)?	Not concerned — Somewhat concerned — Very concerned<br>*(Place a mark on the scale above)*
How concerned are you about exposing others to the coronavirus (COVID-19)?	Not concerned — Somewhat concerned — Very concerned<br>*(Place a mark on the scale above)*
How important is it for you to know if you have previously been exposed to the coronavirus (COVID-19)?	Not important at all — Somewhat important — Very important<br>*(Place a mark on the scale above)*

© 2021 Nelson TL et al. *JAMA Network Open*.

Section IV: Demographic Information and Health History

What is your gender? ◯ Male ◯ Female ◯ Other

Are you Hispanic or Latino? ◯ Yes ◯ No ◯ Prefer not to answer

Which of the following best describes your race (check all that apply)? ☐ White ☐ American Indian or Alaskan Native ☐ Asian ☐ Black or African-American ☐ Naive Hawaiian or other Pacific Islander ☐ Other ☐ Prefer not to answer

If other please specify ________________________________

What is your age? ________________________________

What is your height in feet and inches? ________________________________
(Example: 6' 3" or 6 foot 3 inches If you prefer not to answer type 999)

What is your weight in pounds? ________________________________
(If you prefer not to answer type 999)

Do you have any of the following:

	Yes	No	Do not know
Asthma	◯	◯	◯
Diabetes	◯	◯	◯
History of blood clots	◯	◯	◯
Any form of heart disease	◯	◯	◯
COPD or emphysema	◯	◯	◯
High blood pressure	◯	◯	◯

Do you get at least 150 minutes of moderate exercise (brisk walking, slow biking, dancing) or 75 minutes of vigorous exercise (running/jogging, swimming, basketball, tennis) per week? ◯ Yes ◯ No

Do you currently take any medications? ◯ Yes ◯ No

Please list out medications and dosages if known. ____

Do you regularly get a flu shot? ◯ Yes ◯ No

Do you have allergies? ◯ Yes ◯ No

Please list out any allergies ____

© 2021 Nelson TL et al. *JAMA Network Open*.

Do you take any supplements (such as vitamins, fish oil, etc)?	◯ Yes ◯ No
Please list the supplements and dosages if known.	________________

© 2021 Nelson TL et al. *JAMA Network Open*.
